# Possible roles for polycomb repressive complex 2 in cereal endosperm

**DOI:** 10.3389/fpls.2015.00144

**Published:** 2015-03-12

**Authors:** Kaoru Tonosaki, Tetsu Kinoshita

**Affiliations:** Kihara Institute for Biological Research, Yokohama City UniversityYokohama, Japan

**Keywords:** endosperm, epigenetics, cereal plants, polycomb, imprinting

## Abstract

The polycomb repressive complex 2 (PRC2) is an evolutionarily conserved multimeric protein complex in both plants and animals. In contrast to animals, plants have evolved a range of different components of PRC2 and form diverse complexes that act in the control of key regulatory genes at many stages of development during the life cycle. A number of studies, particularly in the model species *Arabidopsis thaliana,* have highlighted the role of PRC2 and of epigenetic controls via parent-of-origin specific gene expression for endosperm development. However, recent research in cereal plants has revealed that although some components of PRC2 show evolutionary conservation with respect to parent-of-origin specific gene expression patterns, the identity of the imprinted genes encoding PRC2 components is not conserved. This disparity may reflect the facts that cereal plant genomes have undergone different patterns of duplication during evolution compared to *A. thaliana* and that the endosperm development program is not identical in monocots and eudicots. In this context, we focus this review on the expression of imprinted PRC2 genes and their roles in endosperm development in cereals.

## Introduction

The endosperm of plant seeds is the most important tissue in plants with regard to human life, because of its importance as a major source of dietary calories. Recent studies have highlighted the role played by polycomb repressive complex 2 (PRC2) as one of the controlling mechanisms of normal endosperm development (Kohler and Makarevich, 2006; Pien and Grossniklaus, 2007; Holec and Berger, 2012). PRC2 is an evolutionarily conserved, high molecular weight complex that was originally identified in *Drosophila* mutants because of its regulation of body-segmentation during embryogenesis (Pirrotta, 1995). Subsequently, PRC2 was shown to have methyltransferase activity for Lys27 of histone H3 (H3K27; Simon and Kingston, 2009). In *Arabidopsis thaliana*, the complex represses expression of target genes through epigenetic modification of the chromatin, and also controls parent-of-origin specific expression of downstream target genes and of the PRC2 component itself in the endosperm (Gehring, 2013). While most of our understanding of the role of PRC2 comes from studies in the model species *A. thaliana*, recent studies in cereal plants, such as maize, barley and rice, have also provided important insights.

In contrast to animal species, such as *Drosophila*, the components of the PRC2 complexes of plant species show considerable variation. Genome evolution in plants involved the generation of multi-gene families and also whole genome duplications, such as in *A. thaliana,* maize and rice (Spillane et al., 2007; Dickinson et al., 2012). It has been hypothesized that whole genome-duplication may reduce evolutionary forces on duplicated genes, resulting in the accumulation of nucleotide substitutions in genes or gain-of-function changes in expression patterns (Ohno, 1970). Additionally, the relaxation of evolutionary constraints might allow transposon insertion at various sites in genes, leading to their silencing (Lynch and Conery, 2000; Rodin and Riggs, 2003). The latter has been postulated to act as a novel epigenetic control through the process of neofunctionalization (Dickinson et al., 2012; Yoshida and Kawabe, 2013). In this intriguing scenario, genes that show specific expression patterns in the endosperm may be associated with targeted genome-wide DNA demethylation in the central cell of the female gametophyte (Dickinson et al., 2012). Mechanisms for imprinted gene expression have been described in many reports (Gehring, 2013); however, questions regarding the biological relevance of genomic imprinting still remain to be answered. The increased understanding of the role of PRC2 in different plant species should be of value to addressing many of the unanswered questions.

## PRC2 in Cereal Plants

The PRC2 complex of animals has four major components: WD40 protein p55 (p55); Suppressor of Zeste 12 [Su(z)12]; Enhancer of Zeste [E(z)]; and extra sex combs (ESC; Schwartz and Pirrotta, 2013). These four components are conserved in *A. thaliana* and in cereal plants (**Table 1**). Although different combinations of the various subunits of PRC2 play distinct roles during development in *A. thaliana*, here we focus on the complex that determines endosperm fate. This complex has been termed FIS-class PRC2, and is encoded by the genes *Multicopy Suppressors of IRA* *1* (*MSI1*), *Fertilization Independent Seed 2* (*FIS2*), *MEDEA* (*MEA*), and *Fertilization Independent Endosperm* (*FIE*), in *A. thaliana* (Kohler and Makarevich, 2006; Pien and Grossniklaus, 2007; Holec and Berger, 2012). To date, the characteristics of this complex have not been fully elucidated in cereal plants.

**Table 1:** Components of Polycomb repressive complex 2 (PRC2).

Species	PRC2 component			
	SET domein	Zinc finger	WD40	WD40
*Drosophila*	E(z)	Su(z)12	Esc	p55
*Arabidopsis*	MEA^∗^	EMF2	FIE	MSI1
	CLF	VRN2		
	SWN	FIS2^∗^		
Barley	HvSWN	HvEMF2a	HvFIE	?
	?	HvEMF2b		
		HvEMF2c		
Maize	Mez1^∗^	ZmEMF2_1	ZmFIE1^∗^	ZmRBAP3
	Mez2	ZmEMF2_2	ZmFIE2	
	Mez3			
Rice	OsCLF	OsEMF2a	OsFIE1^∗^	OsRBAP3
	OsiEZ1(OsSET1)	OsEMF2b	OsFIE2

*^∗^Maternally expressed imprinted gene.*

### p55

The *Drosophila* *p55* homolog in *A. thaliana*, *MSI1*, has been identified as a component of FIS-class PRC2 (Kohler et al., 2003; Guitton et al., 2004). MSI1 is a WD40 repeat protein; a loss-of-function mutant of MSI1 has been shown to display similar defects in cellularization and over-proliferation of endosperm as FIS-class PRC2 mutants. The *MSI1* homologs of maize (*Zea mays*) and rice (*Oryza sativa*) have been identified (**Table 1**) but have yet to be studied in detail (Hennig et al., 2005).

### Su(z)12

Three *Su(z)12* homologs have been identified in the barley (*Hordeum vulgare*) genome, and are termed *HvSu(z)12a*, *HvSu(z)12b,* and *HvSu(z)12c* (Kapazoglou et al., 2010). All three genes are included in the *Embryonic Flower 2* (*EMF2)* clade by phylogenetic analysis (Kapazoglou et al., 2010). *HvSu(z)12b* transcripts have been detected in all tested tissues and found to increase during seed development. Expression of *HvSu(z)12c* is limited to the young shoots and the developing seed; *HvSu(z)12a* has not been detected in any tested tissue (Kapazoglou et al., 2010). The rice genome has two homologs of *Su(z)12*, named* OsEMF2a* and *OsEMF2b,* that are expressed in a wide range of tissues (Luo et al., 2009). Interestingly, eudicots such as *A. thaliana* have a single copy of *EMF2*, while monocots have two or three EMF2-like genes. This suggests that the *EMF2* gene family in the Poaceae (Gramineae) may have arisen from a recent duplication. No orthologs of *VRN2* or *FIS2* of* A. thaliana* have been identified in cereals (Luo et al., 2009).

### E(z)

Analyses of the barley genome have identified one *E(z)* homolog, termed *HvE(z)*, which is within the *SWINGER* (*SWN*) clade (Kapazoglou et al., 2010). Expression of *HvE(z)* occurs in both vegetative and reproductive tissues, and increases during seed development. The highest levels of *HvE(z)* expression have been found in young shoots (Kapazoglou et al., 2010). In maize, three *E(z)* homologs have been identified, namely, *Mez1*, *Mez2,* and *Mez3* (Springer et al., 2002; Haun et al., 2007). The *Mez1* sequence is similar to that of *CLF*, while *Mez2* and *Mez3* are more closely related to *SWN*. The *Mez2* and *Mez3* genes have high sequence identity, suggesting that they are duplicate genes formed during the paleotetraploid origin of maize (Springer et al., 2002). The three genes are widely expressed throughout the maize life cycle. *Mez1* shows maternal-specific gene expression (imprinted) in the endosperm, but shows bi-allelic (non-imprinted) expression patterns in the embryo (Haun et al., 2007). Three splicing variants are transcribed from the *Mez2* locus and show variations in their transcription among tissues (Springer et al., 2002). Analyses of sequence similarities indicate that the rice genome contains two homologs of *E(z)*, namely, *OsiEZ1*(*OsSET1*) and *OsCLF* (Thakur et al., 2003; Luo et al., 2009). These two rice genes are widely expressed in a range of tissues (Luo et al., 2009). Homologs of *E(z)* in cereal plants fall into the *CLF* and *SWN* clades. The *SWN* clade is specific to flowering plants, while the *CLF* clade also contains homologs from spikemosses (*Selaginella spp.*; Luo et al., 2009). The maize homologs of *E(z)* are more diverse than those of other cereal plants; it seems that the multiplication of homologous genes provided diversity of PRC2 functions in maize. The MEA protein is a core component of FIS-class PRC2, which is related to seed development in *A. thaliana*. However, no *MEA-like* gene has been identified in cereals (Luo et al., 2009).

### ESC

Barley genome sequencing identified a single homolog of *ESC* (Kapazoglou et al., 2010); however, two duplicated genes for FIE-like proteins are present in both maize and rice genomes (Springer et al., 2002; Luo et al., 2009). In barley, *HvFIE* is widely expressed in vegetative and reproductive tissues. Similarly, *ZmFIE2* is expressed in a range of tissues in maize (Springer et al., 2002; Danilevskaya et al., 2003). These various genes are therefore the likely functional orthologs in cereals of *FIE* in* A. thaliana. ZmFIE1* in maize and *OsFIE1* in rice are predominantly expressed in the endosperm, and both display maternal-specific expression patterns (Danilevskaya et al., 2003; Gutierrez-Marcos et al., 2006). In maize, analysis using methylation sensitive restriction enzymes and PCR has shown that genome-wide DNA hypomethylation of the maternally derived genome occurs in the endosperm (Lauria et al., 2004). Related to this finding, differentially methylated regions (DMRs) have been identified that involve hypomethylation of the maternal allele of the *ZmFIE1* and *ZmFIE2* genes (Gutierrez-Marcos et al., 2006). The promoter region of *ZmFIE1* is demethylated in the central cell but not in the sperm cells; this asymmetric pattern of DNA methylation is inherited to the endosperm, where the maternally derived *ZmFIE1* is expressed while the paternally derived allele is silenced. The 5^′^ region of *ZmFIE2* is hypomethylated in many tissues, but subjected to *de novo* DNA methylation only on the paternally derived allele in the endosperm after fertilization. These DMRs may be a mechanism for maternal specific gene expression during early endosperm development (Gutierrez-Marcos et al., 2006). Similarly, transcription of the paternal *OsFIE1* allele during early endosperm development is likely silenced by DNA methylation (Luo et al., 2009; Ishikawa et al., 2011; Zhang et al., 2012). The sequences and expression patterns of maize *ZmFIE1* and rice *OsFIE1* are very similar suggesting an orthologous relationship between these genes. In maize, *ZmFIE1* and *ZmFIE2* are located on different chromosomes (Springer et al., 2002), whereas rice *OsFIE1* and *OsFIE2* are located in the same genomic region on chromosome 8. Phylogenetic analysis of these maize and rice genes suggest that the two maize genomic regions arose from reciprocal deletion of one of the ancestral paralogs during maize genome evolution (Swigonova et al., 2004). The fact that rice *OsFIE1* and *OsFIE2* are closely positioned on the same chromosome suggests they arose through an intraspecies gene duplication event (Luo et al., 2009).

## Roles for PRC2 Complexes in Cereal Endosperm

In a comparison of gene expression patterns in two barley cultivars that have seeds of different sizes, differential expression of *HvFIE* and *HvE(z)* was shown to occur during seed development (Kapazoglou et al., 2010). *HvFIE* expression was found to increase immediately after fertilization in both cultivars, and then to decline in the cultivar producing larger seeds, but to increase in the cultivar with smaller seeds. The expression patterns of *HvFIE* are consistent with the predicted role of PRC2 in cereal plants, namely, the repression of endosperm development. *HvFIE* and *HvE(z)* expression can also be induced by the plant hormone abscisic acid (ABA), which is known to be involved in seed maturation, dormancy, and germination (Kapazoglou et al., 2010). These findings suggest that genes for PRC2 components can act at both earlier and later stages of endosperm development in barley; this may reflect the developmental program of endosperm of cereal species. Although the syncytial phase during early endosperm development is conserved in *A. thaliana* and cereal species, embryonic growth in *A. thaliana* later results in the consumption of the endosperm; by contrast, the endosperm persists in cereals (Sabelli and Larkins, 2009; Dante et al., 2014).

In *A. thaliana,* the imprinted genes *MEA* and *FIS2* encode PRC2 components and are involved in endosperm development through repression of the *AGL62* gene expression that controls the timing of cellularization (Kang et al., 2008; Hehenberger et al., 2012). In contrast to *A. thaliana*, *MEA* and *FIS2* orthologs have not been identified in barley, maize, or rice genomes. In rice, with the exception of *OsFIE1*, genes encoding PRC2 components are widely expressed in a range of tissues. *OsFIE1* shows specific expression in the endosperm and is the only imprinted PRC2 gene in rice endosperm (Luo et al., 2009); the gene is expected to be involved in multiple processes during endosperm development including cellularization. Plants homozygous for the *Osfie1* mutation do not display an obvious endosperm phenotype compared to wild type plants (Luo et al., 2009); by contrast, RNAi transgenic plant lines showed autonomous endosperm development (Li et al., 2014). This outcome may be due to off-target effects of the *OsFIE2* RNAi construct which silenced both *OsFIE1* and *OsFIE2* in the endosperm of the transgenic rice (Li et al., 2014). By contrast, the specific down-regulation of *OsFIE2* by RNAi results in the production of small seeds, which contain shrunken and defective endosperm and a relatively large embryo (Nallamilli et al., 2013). Although a sporophytic effect of the knock-down mutation, due to the dominant nature of RNAi construct, cannot be discounted in the latter experiment, this result suggests *OsFIE2* has a positive regulatory role in either early or late development of rice endosperm, in contrast to the role of FIS-class PRC2 in the endosperm of *A. thaliana*. It should be possible to more clearly determine the role of *OsFIE2* through use of the appropriate mutant alleles in combination with TALLEN or CRISPER/Cas technology (Kim and Kim, 2014). Such analyses would elucidate the role of OsFIE2 in endosperm development, especially in relation to the timing of cellularization. There is evidence from interspecific and interploidy crosses in rice that the timing of cellularization and the eventual size of the endosperm are related (Ishikawa et al., 2011; Sekine et al., 2013). Therefore, investigation of cellularization in PRC2 mutants will be an essential approach to understanding the action of PRC2 in cereal endosperm.

**FIGURE 1:**
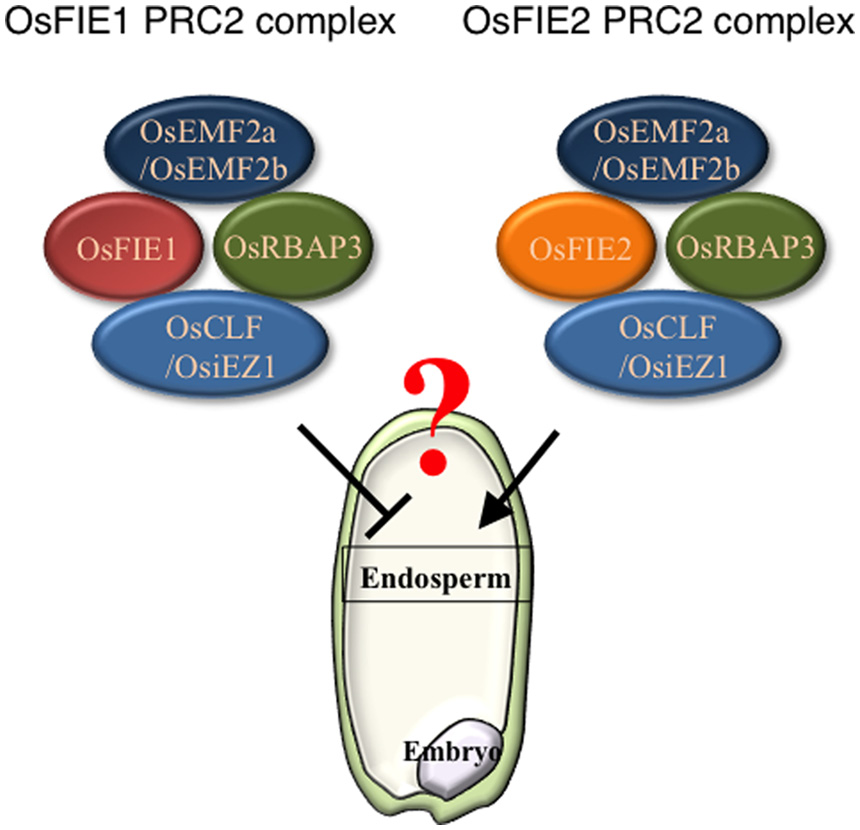
Polycomb repressive complex 2 (PRC2) components of OsFIE1 and OsFIE2 may have distinct roles in rice endosperm. Based on recent findings, PRC2 complexes that contain OsFIE1 and OsFIE2 are likely to have distinct roles. In the endosperm, the OsFIE1 protein is produced from the maternally derived allele and contributes to the FIE1-containing PRC2 (left), By contrast, OsFIE2 protein derived from both maternal and paternal alleles is used to form FIE2-containing PRC2 (right).

Recently, an epigenetic allele of *Epi-df* was identified; this allele is a gain-of-function variant that likely resulted from hypomethylation of the 5^′^ region of *OsFIE1* without any change in nucleotide sequence (Zhang et al., 2012). On the *Epi-df* mutant background, *OsFIE1* is ectopically expressed in vegetative tissues and the normally silent paternally derived allele is active in the endosperm (Zhang et al., 2012). The *Epi-df* plants show dwarfism and floral organ defects in a dominant fashion; the latter prevented investigation of the endosperm phenotype. By contrast, a recent study showed that expression of *OsFIE1* is correlated with the timing of cellularization (Folsom et al., 2014). Under moderately high temperature conditions, *OsFIE1* expression increases, and this elevated level of expression is correlated with precocious endosperm cellularization (Folsom et al., 2014). Similarly, overexpression of *OsFIE1* causes decreased seed sizes and weights (Folsom et al., 2014). This is in contrast with the outcome of *OsFIE2* overexpression, which does not result in phenotypic changes in plants (Nallamilli et al., 2013). Overall, these findings suggest the possibility that *OsFIE1* and *OsFIE2* may have non-equivalent roles in endosperm development (**Figure 1**). Further analyses will be required to clarify precisely the roles of PRC2 in the cereal endosperm development.

## Conclusion

The data generated by cereal genome sequencing initiatives have enabled the identification of PRC2 genes in crop plant species. Detailed analyses of the expression of these genes have revealed remarkable differences in their behavior compared to orthologs in *A. thaliana*. Endosperm specific variants of the *Su(Z)12* homolog and *E(z)* homolog have been found, namely, *MEA* and *FIS2*; however, no variants of the *ESC* homolog are known in *A. thaliana*. By contrast, two *ESC* homologs *FIE1* and *FIE2* are present in maize and rice genomes. Although *FIE* is not consistently imprinted in *A. thaliana* (Yadegari et al., 2000), its homologs in maize and rice show maternal specific expression (Gutierrez-Marcos et al., 2006; Luo et al., 2009). In general, ESC and its homologs are WD40 repeat scaffolding proteins and do not seem to have any enzymatic activity. However, their animal counterparts have been shown to have binding activity for the N-terminal histone tail of H3 and to cause allosteric effects on the histone methyltransferase activity of EZH2; binding to chromatin residues associated with a repressive state of gene expression, such as H3K9me3, induces histone methyltransferase activity, while binding to chromatin residues associated with active transcription reduces its activity. Therefore, the protein–protein interactions of each PRC2 component are important determinants of the activity of the PRC2 complex. Further study of cereal PRC2 complexes will undoubtedly provide greater insights into their roles in endosperm development.
